# Supplementary material for: Reanalysis of ribosome profiling datasets reveals a function of rocaglamide A in perturbing the dynamics of translation elongation via eIF4A
Source: Nat Commun. 2023 Feb 2;14:553. doi: 10.1038/s41467-023-36290-w (PMC9891901; doi:10.1038/s41467-023-36290-w)
Supplement: Supplementary file 1 — Supplementary Information [file 41467_2023_36290_MOESM1_ESM.pdf]

Supplementary Information

Reanalysis of ribosome profiling datasets reveals a novel function of rocaglamide A in perturbing the dynamics of translation elongation via eIF4A

Fajin Li[1,2,3,$,*], Jianhuo Fang[1,2,$], Yifan Yu[1,2,$], Sijia Hao[1,2,3], Qin Zou[1,2], Qinglin Zeng[1,2], and Xuerui Yang[1,2,3,*]

[1] MOE Key Laboratory of Bioinformatics, School of Life Sciences, Tsinghua University, Beijing 100084, China

[2] Center for Synthetic & Systems Biology, Tsinghua University, Beijing 100084, China

[3] Joint Graduate Program of Peking-Tsinghua-National Institute of Biological Science, Tsinghua University, Beijing 100084, China.

[$] These authors contributed equally to this work.

*Correspondence to: Fajin Li (lfj17@tsinghua.org.cn) and Xuerui Yang (yangxuerui@tsinghua.edu.cn)

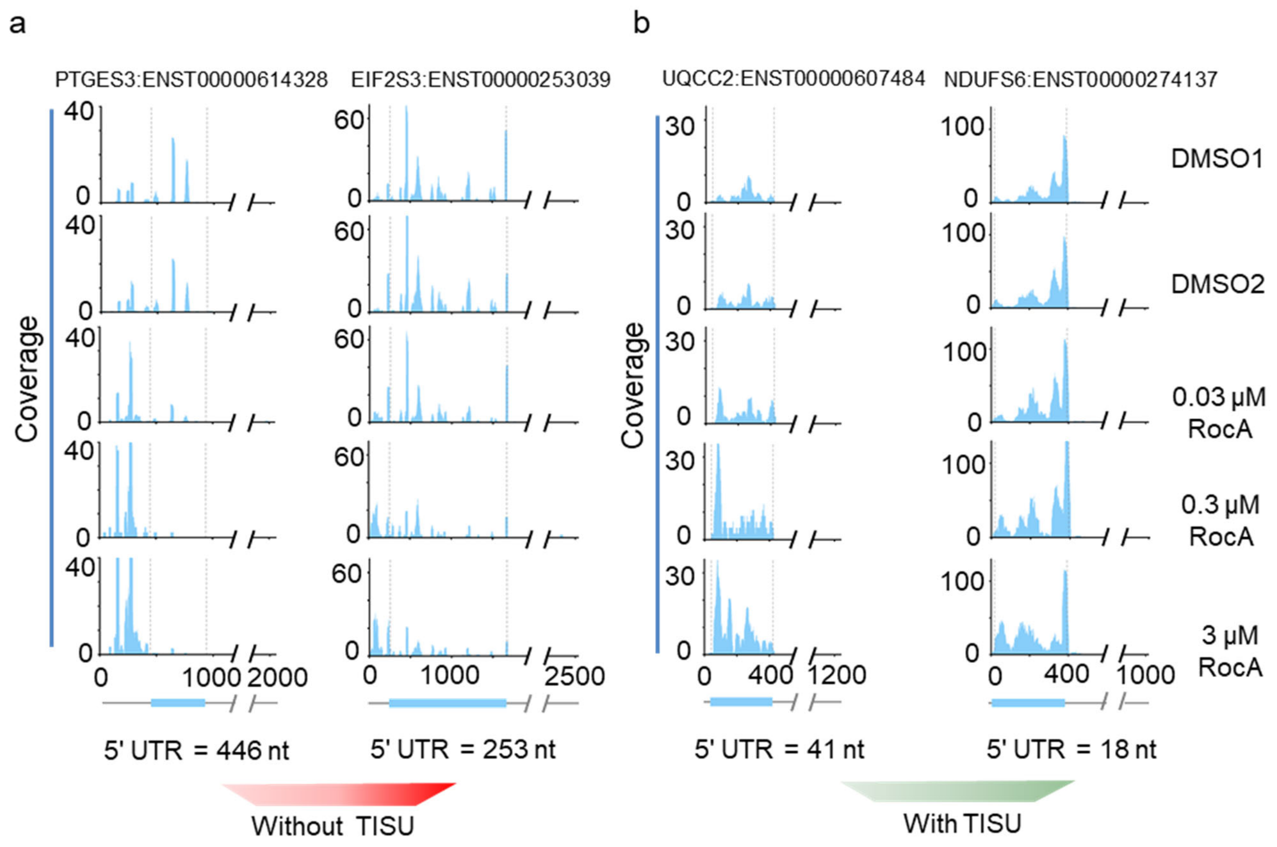

Supplementary Figure 1. Examples of ribosome accumulation in 5' UTR and CDS regions induced by RocA treatments. a) RPF coverages of PTGES3 and EIF2S3 as examples without TISU element in 5' UTRs. **b)** RPF coverages of UQCC2 and NDUFS6 as examples with TISU element in 5' UTRs.

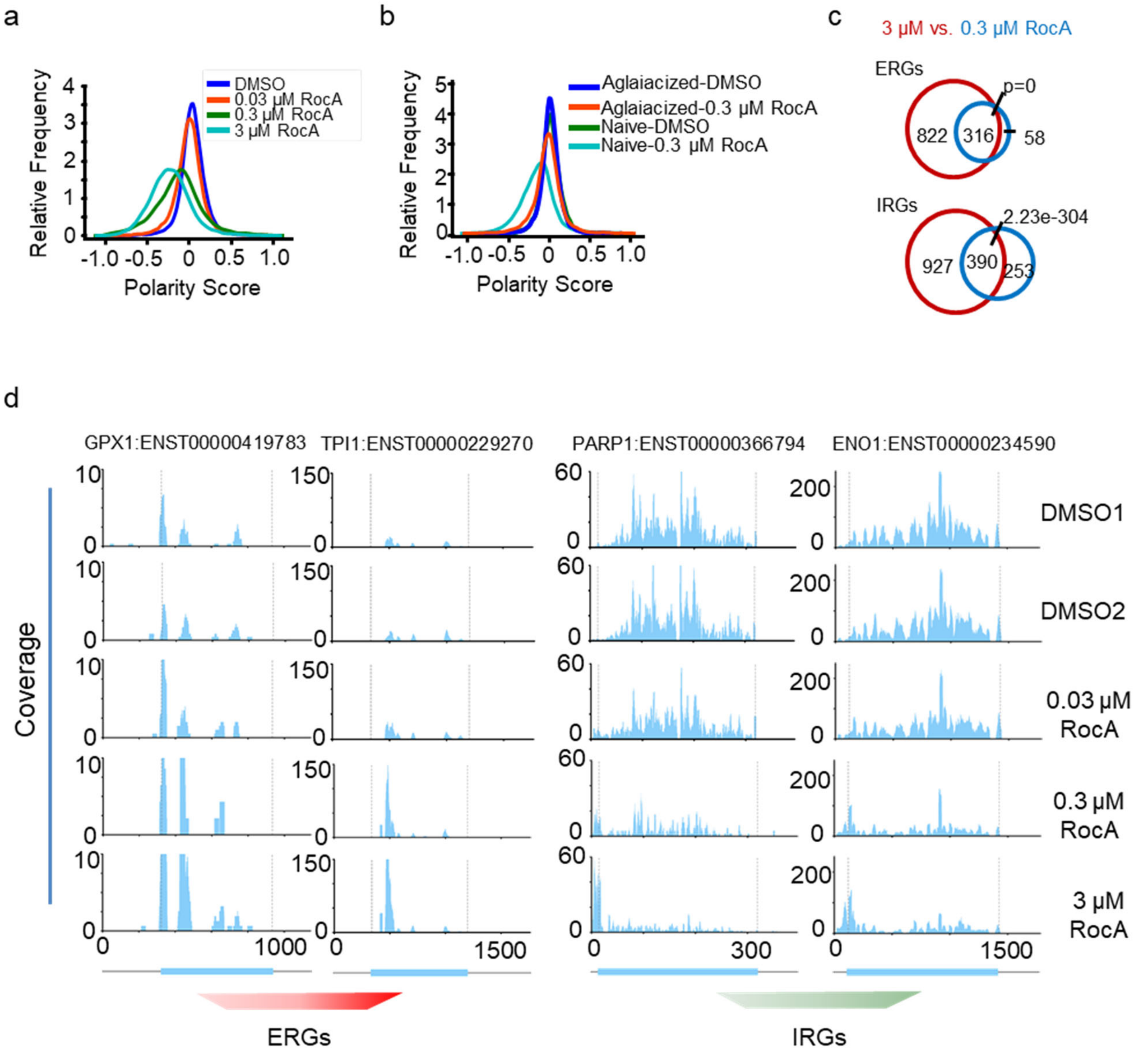

Supplementary Figure 2. Gene-specific patterns of ribosome distribution shifts induced by RocA. a) Distributions of the polarity scores of all the genes under different conditions in HEK293 cells. The numbers of transcripts used for the plots are consistent to those in Figure 1B. **b)** Distributions of the polarity scores of all the genes under different conditions in normal (Naïve) or HEK293 cells with eIF4A1 double mutations (Phe163Leu-Ile199Met, Aglaiacized). **c)** Venn plots showing overlaps between ERGs or IRGs identified in cells treated with different doses of RocA. The P-values are calculated with the hypergeometric test. **D)** RPF coverages of two IRGs (PARP1 and ENO1) and two ERGs (GPX1 and TPI1) as examples. Source data are provided as a Source Data file.

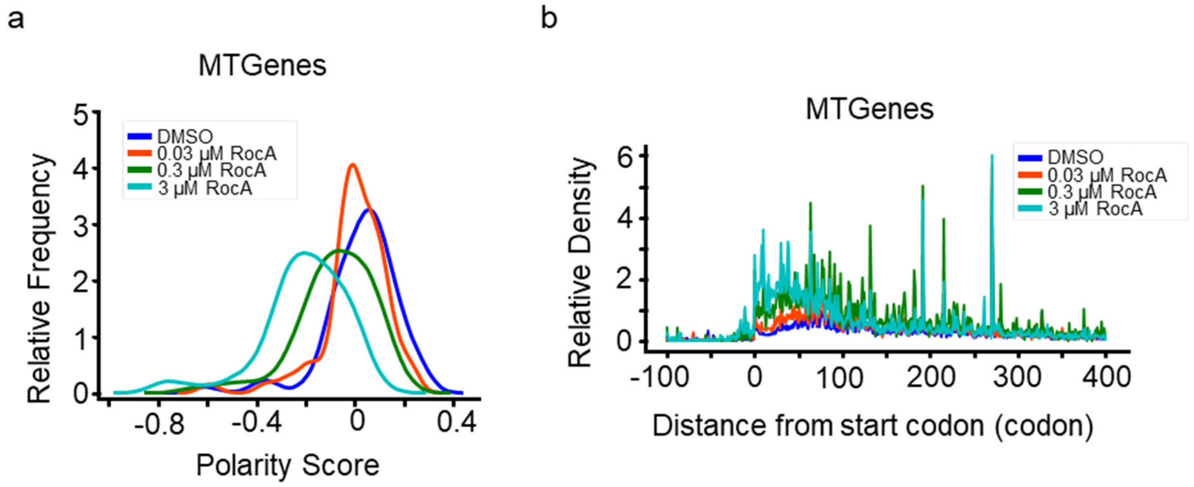

Supplementary Figure 3. Ribosome distributions of the mitochondrial function associated genes (MTgenes). a) Distributions of the polarity scores of 64 MTGenes under different conditions in HEK293 cells. **b)** Metagene plots of the averaged RPF read densities for the 64 MTGenes. The X-axis represents the distance from the start codon. Source data are provided as a Source Data file.

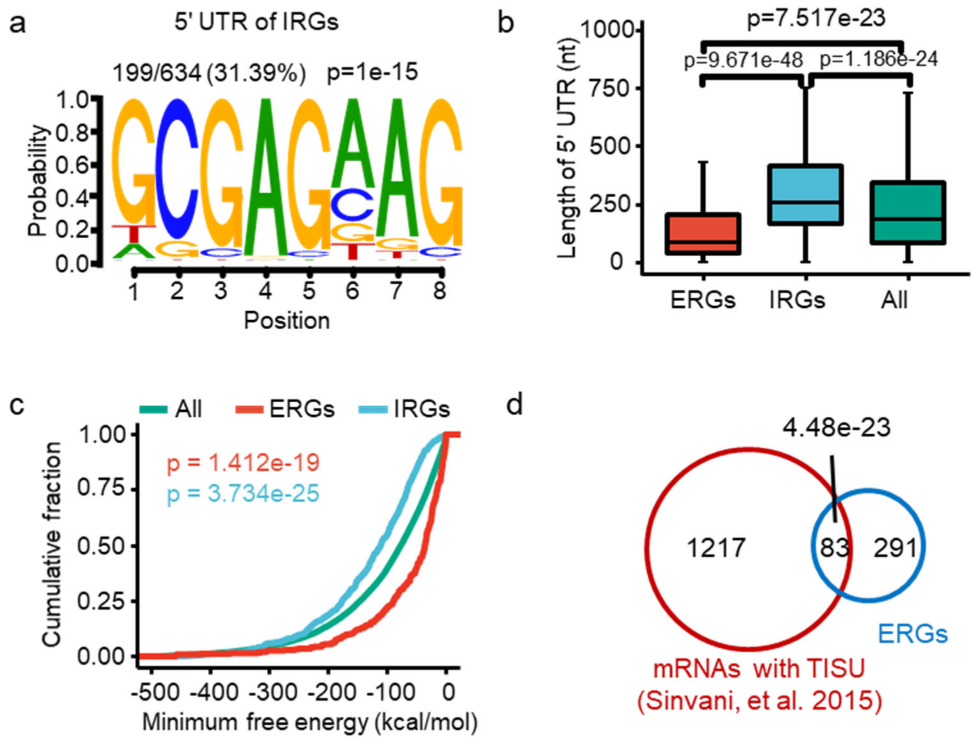

Supplementary Figure 4. Sequence features of ERGs and IRGs. a) Poly-purine motifs enriched in the 5'UTRs of IRGs. **b)** Boxplots showing length distributions of the 5'UTRs for ERGs and IRGs. Transcripts used for statistics: All (n=19639), ERGs (n=374), IRGs (n=643). P-values were calculated by two-sided Wilcoxon rank sum test. Center line, median; box limits, upper and lower quartiles; whiskers, 1.5x interquartile range; outliers were not shown here. **c)** Cumulative density plots showing distributions of the minimum free energy of the 5'UTRs. P-values were calculated by two-sided Wilcoxon rank sum test. **d)** Venn plot showing the overlap between ERGs and the mRNAs with TISU elements identified by Sinvani, et al (Sinvani et al., 2015). Source data are provided as a Source Data file.

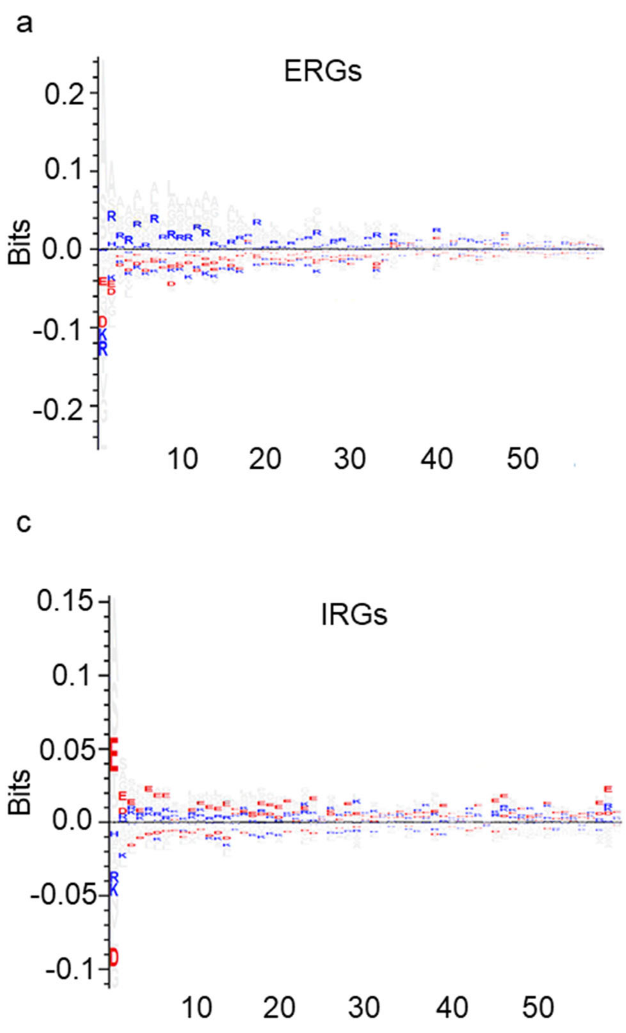

Supplementary Figure 5. Amino acid sequence features of ERGs and IRGs. a-c) Sequence logos showing enrichments of the first 55 amino acids of ERGs, IRGs and MTGenes. Amino acids with positive charges are shown in blue and negative charges in red.

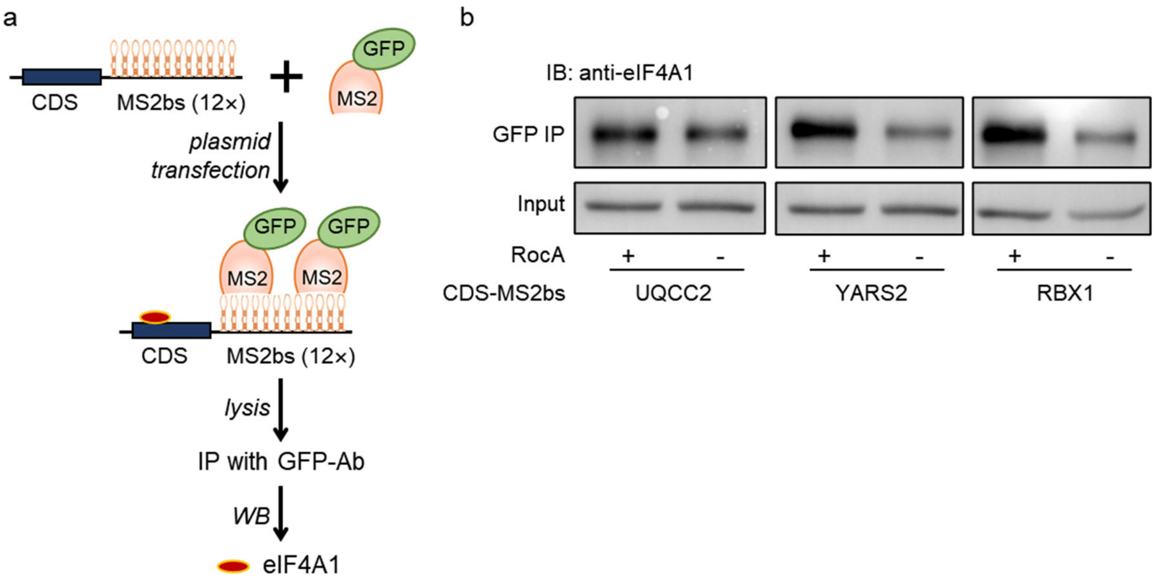

Supplementary Figure 6. MS2-GFP-RIP assays with CDS regions of ERGs. (a) Schematic description of the MS2-GFP-RIP with the CDS regions of 3 ERGs. **(b)** MS2-GFP-RIP assay by immunoprecipitation of GFP, followed by immunoblotting of eIF4A1. Source data are provided as a Source Data file.

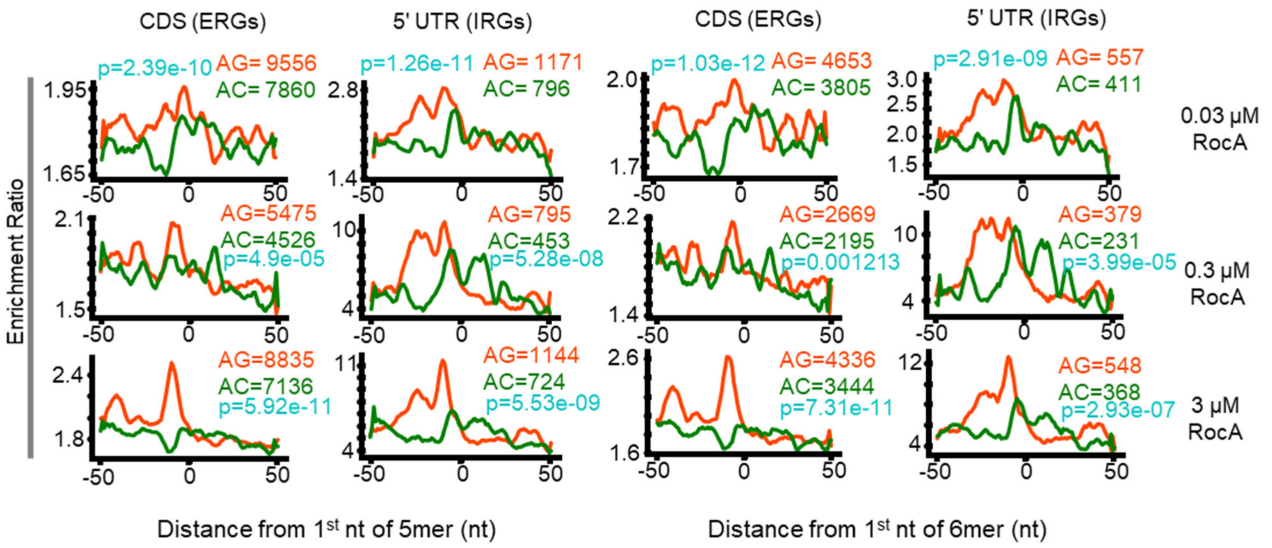

Supplementary Figure 7. Enrichment of ribosome density around the poly purine motifs of ERGs and IRGs in response to RocA. Enrichment ratio of ribosome density around poly-purine (poly-AG) and poly-AC motifs (5-mer and 6-mer) in the 5' UTR of IRGs and CDS of ERGs based on comparisons between RocA and DMSO. P-values were calculated by two-sided Student's t test.

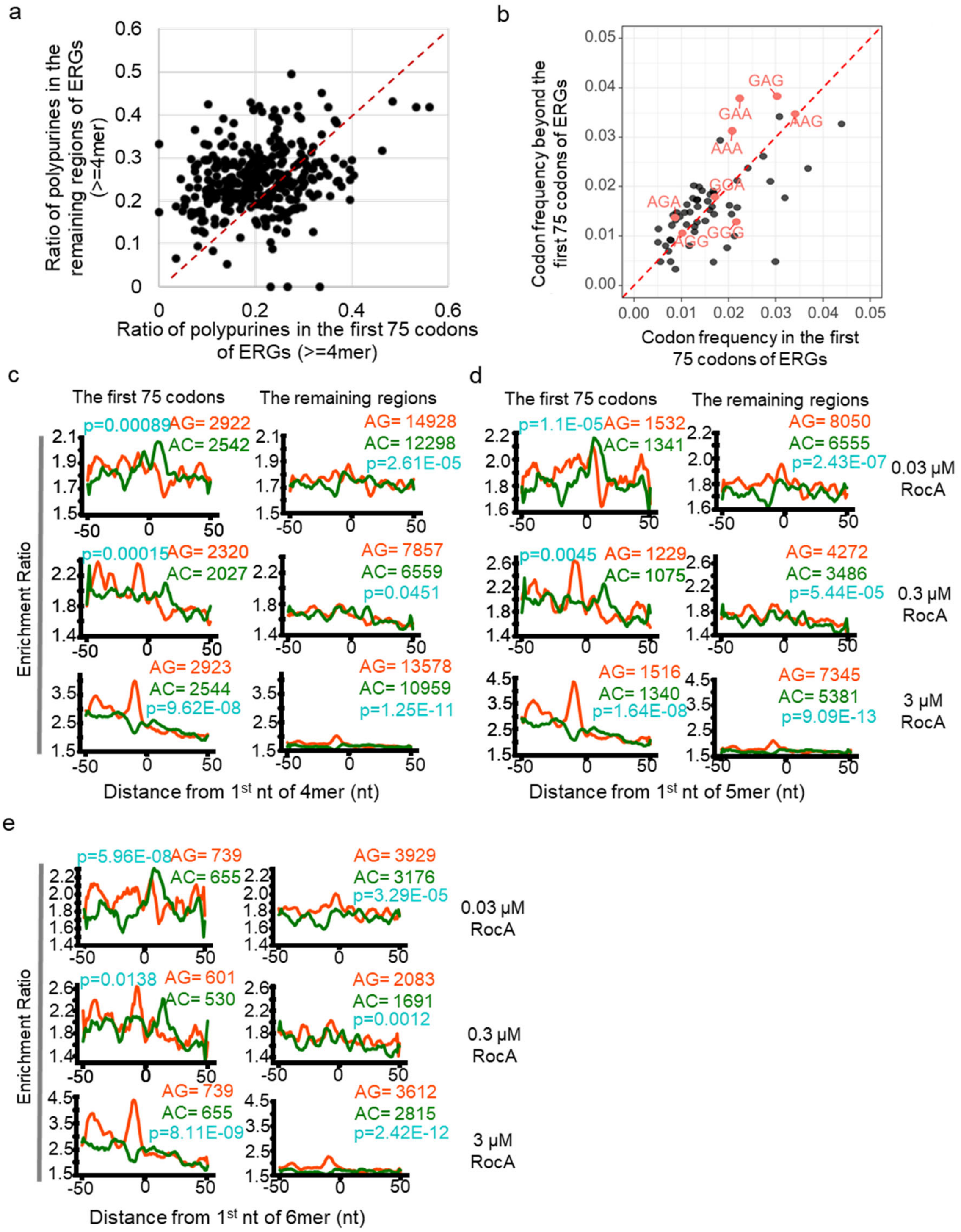

Supplementary Figure 8. Positional effects of poly-purine motifs in the CDS of ERGs. a) Enrichments of poly-purine sequences in the first 75 codons and after the first 75 codons. **b)** Enrichments of AG-rich codons in the first 75 codons and after the first 75 codons. **c-e)** Enrichment ratio of ribosome density around the poly purine motifs in the first 75 codons of the ERGs and in the regions beyond the first 75 codons of ERGs in response to RocA; P-values are all calculated by two-sided Student's t test without adjustments. Source data are provided as a Source Data file.

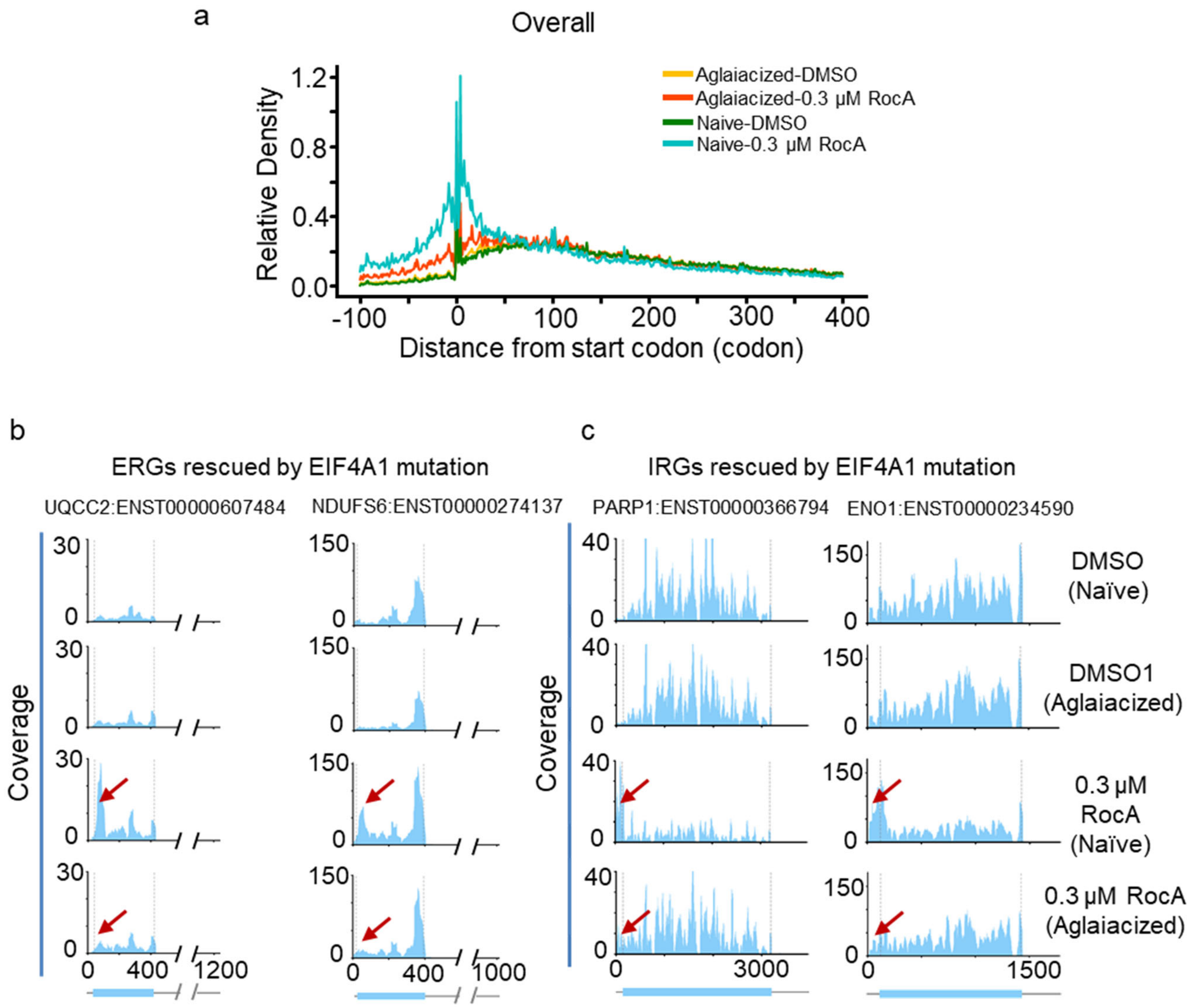

Supplementary Figure 9. Ribosome distributions in the HEK293 cells bearing EIF4A1 double mutations. a) Metagene plots of the averaged RPF read densities for all the genes. The X-axis represents the distance from the start codon. Naïve, normal HEK293 cell. Aglaiacized, HEK293 cell with EIF4A1 double mutations. Numbers of the genes used for the plots are 10043 (Naïve-DMSO), 8606 (Naïve-RocA03), 9899 (Aglaiacized-DMSO) and 9187 (Aglaiacized-RocA03). **b, c)** RPF coverages of two ERGs (UQCC2 and NDUFS6) and two IRGs (PARP1 and ENO1) as examples in the cells with EIF4A1 double mutations. Red arrows indicate where ribosome stalling takes place in normal cells upon RocA treatment but not in cell bearing eIF4A1 double mutations. Source data are provided as a Source Data file.

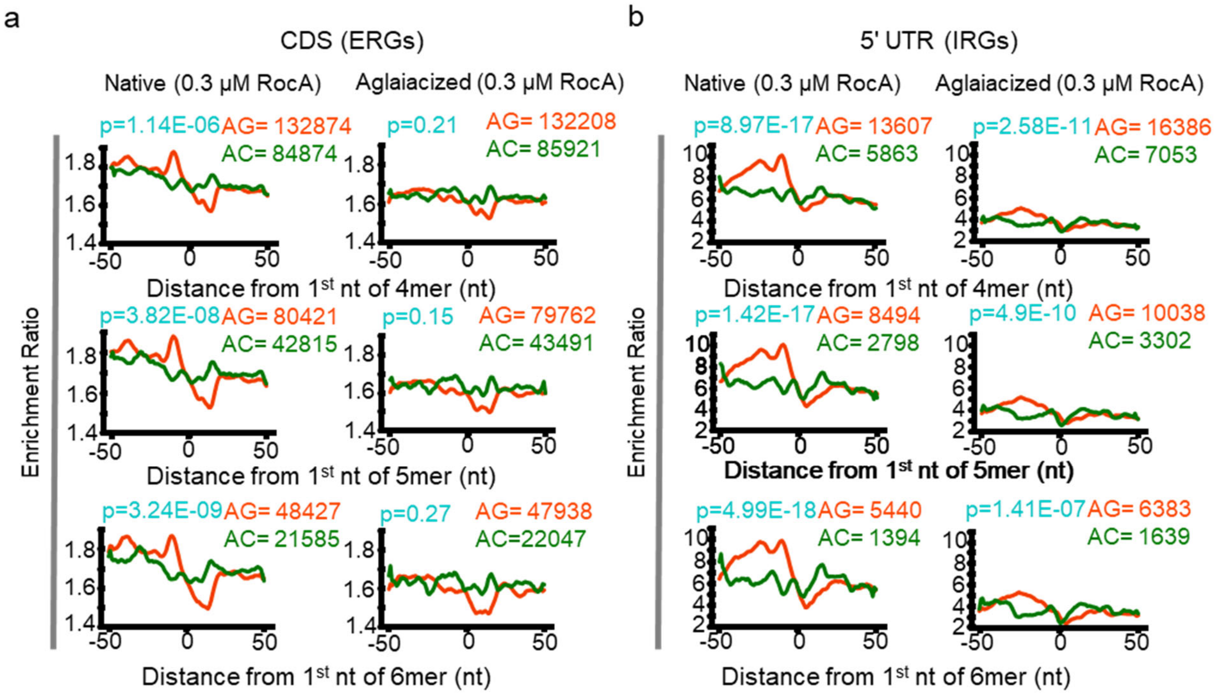

Supplementary Figure 10. Enrichment of ribosome density around the poly purine motifs for both ERGs and IRGs in response to RocA. Enrichment ratio of ribosome density around poly-purine (poly-AG) and poly-AC motifs (4-mer, 5-mer and 6-mer) in the CDS of ERGs (A) or in the 5' UTR of IRGs (B) based on comparisons between RocA and DMSO after eIF4A1's double mutations. Naïve, normal HEK293 cell. Aglaiacized, HEK293 cell with EIF4A1 double mutations. P-values are all calculated by two-sided Student's t test without adjustments.

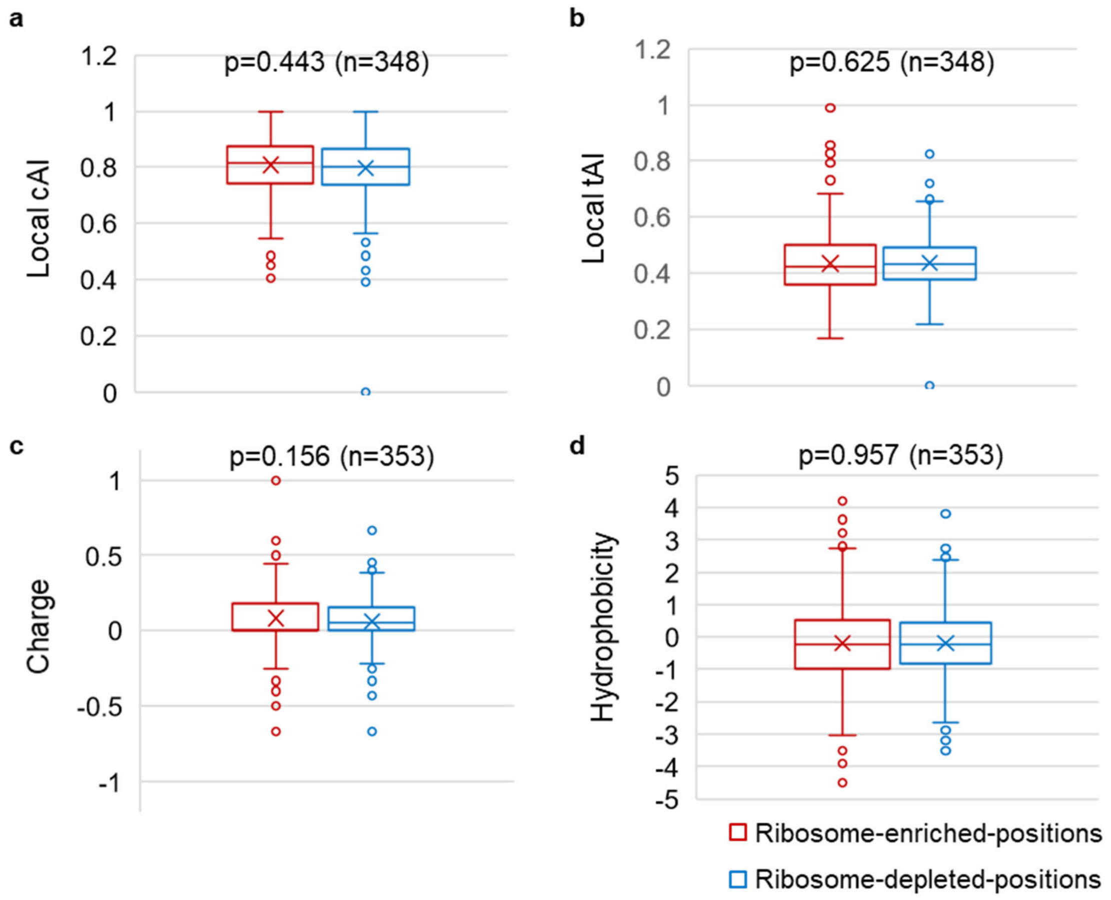

Supplementary Figure 11. Sequences features of ribosome-enriched-positions and ribosome-depleted-positions. a-d) comparisons of the local cAI, the local tAI, average charge of amino acids, and hydrophobicity of amino acids of sequences in ribosome-enriched-positions and ribosome-depleted-positions for each transcript. The mean feature scores in ribosome-enriched-positions and ribosome-depleted-positions of each transcript are calculated, respectively. Significance was calculated by two-sided Student's t test. "n=348, n=353" represents the final transcripts used for statistics. Center line, median; box limits, upper and lower quartiles; whiskers, 1.5x interquartile range; points, outliers; "x", mean. Source data are provided as a Source Data file.

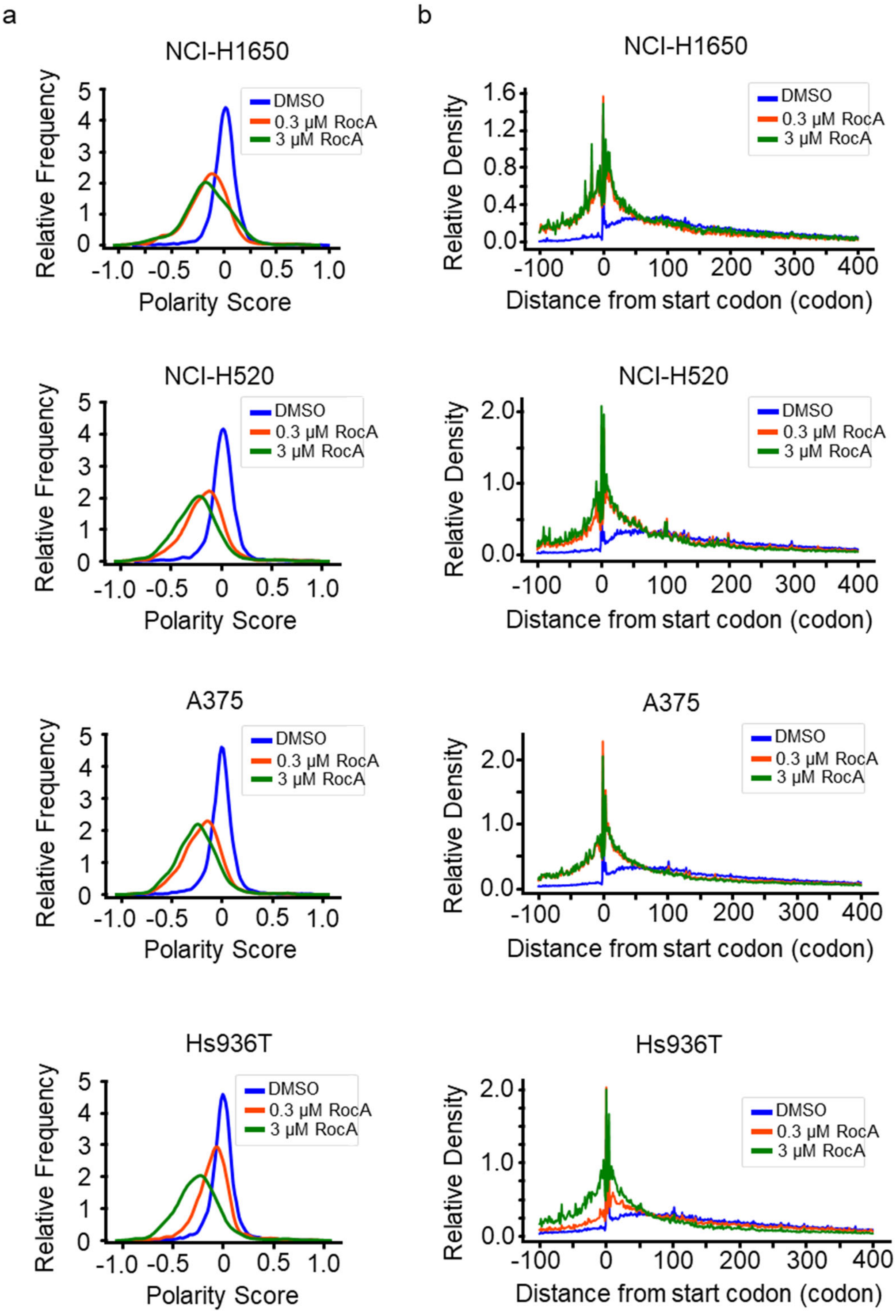

Supplementary Figure 12. Ribosome distributions shifted by RocA in lung cancer and melanoma cell lines. a) Distributions of the polarity scores of in 4 cancer cell lines treated with two concentrations of RocA. **b)** Metagene plots of the averaged RPF read densities in 4 cancer cell lines treated with RocA. The X-axis represents the distance from the start codon. Source data are provided as a Source Data file.

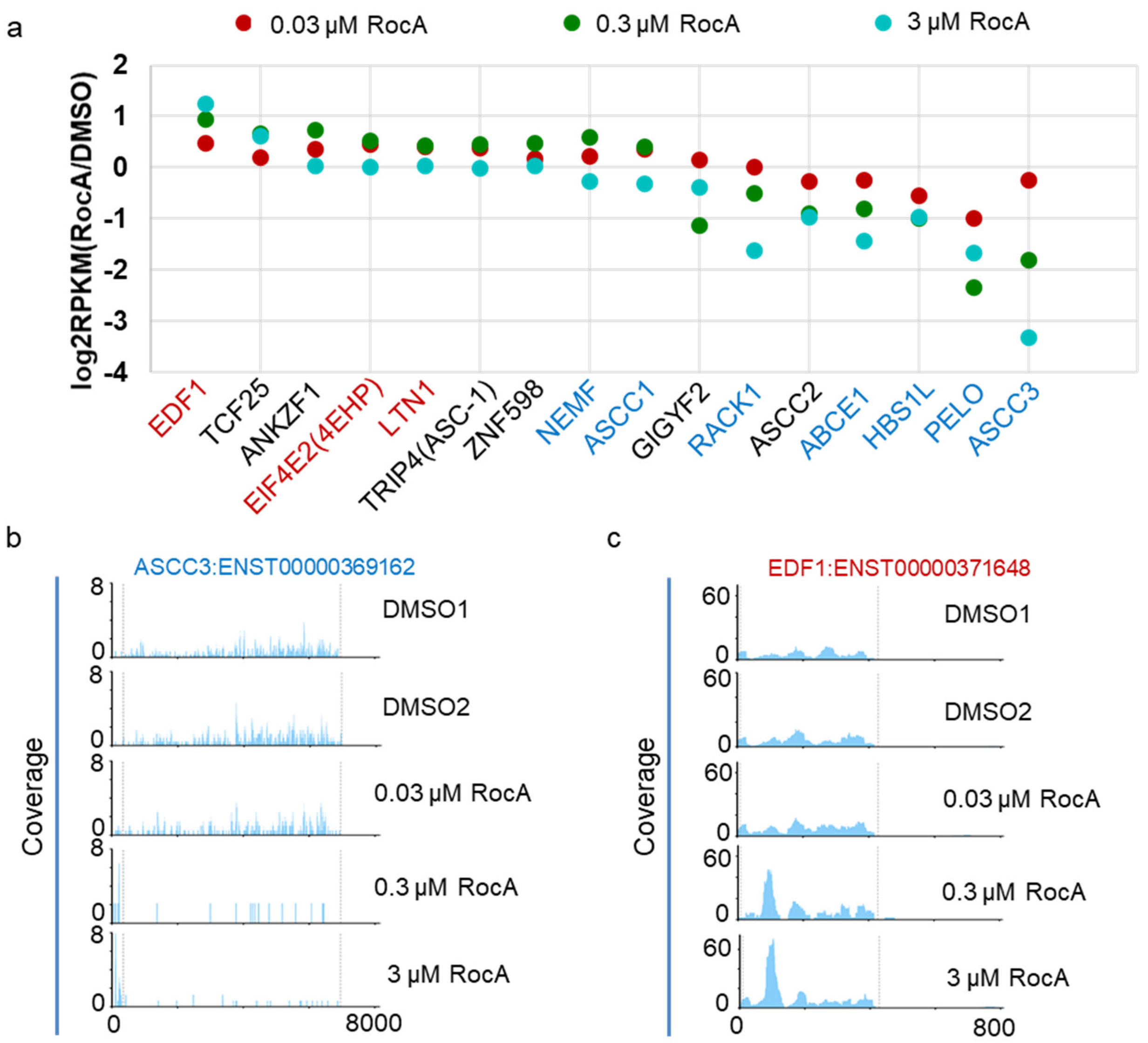

Supplementary Figure 13. Perturbed translation of the RQC genes upon RocA treatment. a) Fold changes of the RPF counts (RPKM) for the RQC genes upon treatments of RocA at different concentrations. ERGs candidates are marked in red and IRGs in blue. **b-c)** RPF coverages of RQC genes, EDF1 as an example ERGs (c) and ASCC3 as an example of IRGs (b), in normal HEK293 cells. Source data are provided as a Source Data file.
