# Supplementary material for: Reanalysis of ribosome profiling datasets reveals a function of rocaglamide A in perturbing the dynamics of translation elongation via eIF4A
Source: Nat Commun. 2023 Feb 2;14:553. doi: 10.1038/s41467-023-36290-w (PMC9891901; doi:10.1038/s41467-023-36290-w)
Supplement: Supplementary file 3 — Description of Additional Supplementary Files [file 41467_2023_36290_MOESM3_ESM.pdf]

Description of Additional Supplementary Files

File Name: Supplementary Data 1
Description: Information of the public datasets used in the present study.

File Name: Supplementary Data 2
Description: ERGs and IRGs identified in the present study.

File Name: Supplementary Data 3
Description: ERGs with TISU and mitochondrial genes.

File Name: Supplementary Data 4
Description: Sequences inserted into the luciferase reporter plasmids.
