# Supplementary material for: Reanalysis of ribosome profiling datasets reveals a function of rocaglamide A in perturbing the dynamics of translation elongation via eIF4A
Source: Nat Commun. 2023 Feb 2;14:553. doi: 10.1038/s41467-023-36290-w (PMC9891901; doi:10.1038/s41467-023-36290-w)
Supplement: Supplementary file 8 — Reporting Summary [file 41467_2023_36290_MOESM8_ESM.pdf]

nature portfolio

Corresponding author(s): Xuerui Yang

Last updated by author(s): Dec 18, 2022

Reporting Summary

Nature Portfolio wishes to improve the reproducibility of the work that we publish. This form provides structure for consistency and transparency in reporting. For further information on Nature Portfolio policies, see our Editorial Policies and the Editorial Policy Checklist.

Statistics

For all statistical analyses, confirm that the following items are present in the figure legend, table legend, main text, or Methods section.

n/a	Confirmed	
☐	☒	The exact sample size (*n*) for each experimental group/condition, given as a discrete number and unit of measurement
☐	☒	A statement on whether measurements were taken from distinct samples or whether the same sample was measured repeatedly
☐	☒	The statistical test(s) used AND whether they are one- or two-sided<br>*Only common tests should be described solely by name; describe more complex techniques in the Methods section.*
☒	☐	A description of all covariates tested
☐	☒	A description of any assumptions or corrections, such as tests of normality and adjustment for multiple comparisons
☐	☒	A full description of the statistical parameters including central tendency (e.g. means) or other basic estimates (e.g. regression coefficient) AND variation (e.g. standard deviation) or associated estimates of uncertainty (e.g. confidence intervals)
☐	☒	For null hypothesis testing, the test statistic (e.g. *F*, *t*, *r*) with confidence intervals, effect sizes, degrees of freedom and *P* value noted<br>*Give P values as exact values whenever suitable.*
☒	☐	For Bayesian analysis, information on the choice of priors and Markov chain Monte Carlo settings
☐	☒	For hierarchical and complex designs, identification of the appropriate level for tests and full reporting of outcomes
☒	☐	Estimates of effect sizes (e.g. Cohen's *d*, Pearson's *r*), indicating how they were calculated

Our web collection on statistics for biologists contains articles on many of the points above.

Software and code

Policy information about availability of computer code

Data collection	All raw fastq files of Ribo-seq and iCLIP-seq data were downloaded from GEO database with fastq-dump software (v2.8.1)
Data analysis	All the Ribo-seq datasets we collected were pre-processed with a snakemake (v5.31.1) pipeline we developed, including adapter trimming, quality filtering, removing rRNA contaminations, mapping, 3-nt periodicity checking, read length distribution,et al. RiboMiner (v0.2.3.2)was used for calculating the polarity scores, metagene analyses and computing read density of a single transcript. All other custom scripts used for data analyses and a detailed description of how to re-generate the main results presented in the manuscript were all deposited in the github (https://github.com/sherkinglee/RocA). Other softwares and tools we used in the present study include bowtie v1.1.2, STAR v2.7.7a, samtools v1.11, sra-tools v2.11, fastx_toolkit v0.0.14, cutadapt v3.1, RiboCode, R >=3.6, Rstudio and python >=3.6, Micosoft Excel, HOMER, Metascape, clusterProfiler v4.6, ViennaRNA v2.5, et al.

For manuscripts utilizing custom algorithms or software that are central to the research but not yet described in published literature, software must be made available to editors and reviewers. We strongly encourage code deposition in a community repository (e.g. GitHub). See the Nature Portfolio guidelines for submitting code & software for further information.

Data

Policy information about availability of data

All manuscripts must include a data availability statement. This statement should provide the following information, where applicable:

- Accession codes, unique identifiers, or web links for publicly available datasets
- A description of any restrictions on data availability
- For clinical datasets or third party data, please ensure that the statement adheres to our policy

Information of all the public ribosome profiling and iCLIP-seq datasets used in the present study is provided in Table S1. The Disome-seq data obtained in this study has been deposited into the GEO database: GSE201364.

Human research participants

Policy information about studies involving human research participants and Sex and Gender in Research.

Reporting on sex and gender	There are no human research participants.
Population characteristics	There are no human research participants.
Recruitment	There are no human research participants.
Ethics oversight	There are no human research participants.

Note that full information on the approval of the study protocol must also be provided in the manuscript.

Field-specific reporting

Please select the one below that is the best fit for your research. If you are not sure, read the appropriate sections before making your selection.

☒ Life sciences ☐ Behavioural & social sciences ☐ Ecological, evolutionary & environmental sciences

For a reference copy of the document with all sections, see nature.com/documents/nr-reporting-summary-flat.pdf

Life sciences study design

All studies must disclose on these points even when the disclosure is negative.

Sample size	There was a total of 24 human Ribo-seq datasets from different labs with more than 100 samples, covering many known inhibitors of translation initiation and elongation, which were all with a good 3-nt periodicity and relatively high sequencing depth. Those Ribo-seq datasets consisted 49 "treatment vs. control" condition pairs, which could be well used for comparisons of the ribosome distribution of each transcript under different conditions and assessing the effects of different conditions on the ribosome distribution of the CDS regions. A set of iCLIP-seq data of eIF4A1 under rocaglamide A (RocA) treatment with distinct concentrations was collected for identifying the binding sequences of eIF4A1 after RocA treatment. And we also generated a set of Disome-seq data under RocA treatment for identifying the ribosome collisions induced by RocA. Ribo-seq datasets of 4 tumor cells under 0.3 µM and 3 µM RocA treatment were collected for assessing whether there was a relationship between its anti-tumor activity and its inhibiting effect on translation elongation.
Data exclusions	No data was excluded from the analyses.
Replication	Most of the public Ribo-seq dataset we collected has at least 2 replicates. The iCLIP-seq samples of DMSO has 2 replicates and RocA samples have no replicates but have 3 different concentrations. The Disome-seq samples of RocA has 2 replicates.
Randomization	The Ribo-seq datasets we collected were divided into 2 groups based on whether it could inhibit translation initiation or elongation and RocA treatment was another group.
Blinding	Blinding was not relevant as no direct comparison between experimental groups was done. The study is mainly analysis of the public data.

Reporting for specific materials, systems and methods

We require information from authors about some types of materials, experimental systems and methods used in many studies. Here, indicate whether each material, system or method listed is relevant to your study. If you are not sure if a list item applies to your research, read the appropriate section before selecting a response.

March 2021

Materials & experimental systems

n/a	Involved in the study
☐	☒ Antibodies
☐	☒ Eukaryotic cell lines
☒	☐ Palaeontology and archaeology
☒	☐ Animals and other organisms
☒	☐ Clinical data
☒	☐ Dual use research of concern

Methods

n/a	Involved in the study
☒	☐ ChIP-seq
☒	☐ Flow cytometry
☒	☐ MRI-based neuroimaging

Antibodies

Antibodies used	anti-GFP: 3µg per reaction, abcam, cat # ab290; anti-eIF4A: 1:1000 dilution, Biorbyt, cat # orb353605.
Validation	anti-GFP: validated by producer, suitable for: WB, ICC/IF. anti-eIF4A: validated by producer, suitable for IF.

Eukaryotic cell lines

Policy information about cell lines and Sex and Gender in Research

Cell line source(s)	HEK293 cell line from ATCC
Authentication	Authenticated by ATCC with STR profiling (Amelogenin: X; CSF1PO: 11,12; D13S317: 12,14; D16S539: 9,13; D5S818: 8,9; D7S820: 11,12; THO1: 7,9.3; TPOX: 11; vWA: 16,19)
Mycoplasma contamination	All the cells were tested for mycoplasma contamination every 3 months, and the results have been negative.
Commonly misidentified lines (See ICLAC register)	No commonly misidentified cell lines were used in the study.
